# A nurse-inserted peripherally inserted central catheter program in general pediatrics: a single-center experience

**DOI:** 10.1186/s12887-022-03809-x

**Published:** 2023-01-14

**Authors:** Zhuowen Yu, Xiaojing Hu, Xiaofeng Xu, Lili Lin, Ying Gu, Jianguo Zhou

**Affiliations:** 1 Department of Gastroenterology and Pulmonology, Children’s Hospital of Fudan University, Shanghai, China; 2 Nursing department office, Children’s Hospital of Fudan University, Shanghai, China; 3 Department of Neonatology, Children’s Hospital of Fudan University, Shanghai, China

**Keywords:** Peripherally inserted central catheter, Pediatrics, Malposition, Complication, arm movements

## Abstract

**Background:**

A peripherally inserted central catheter (PICC) with its tip preferably in the vena cava is essential in caring for patients with chronic conditions in general pediatrics. However, PICC-related complications are concerning and warrant further investigations.

**Objectives:**

To share the experience of a nurse-inserted peripherally inserted central catheters (PICC) program initiated in a general pediatric department**.**

**Methods:**

A retrospective descriptive cohort study based on a prospectively collected database was conducted. All PICCs inserted in the departments of gastroenterology and pulmonology in a tertiary pediatric center from Dec. 2015 to Dec. 2019 were included in the study. Complications and risk factors were analyzed by comparing cases with and without complications. We also reported arm movements in correcting mal-positioned newly-inserted PICCs.

**Results:**

There were 169 cases with a median (IQR) age of 42(6, 108) months who received PICC insertion during a 4-year period. Inflammatory bowel disease was the leading diagnosis accounting for 25.4% (43/169) of all cases. The overall complication rate was 16.4 per 1000 catheter days with malposition and occlusion as the two most common complications. Multivariate models performed by logistic regression demonstrated that young age [*p* = 0.004, OR (95%CI) = 0.987(0.978, 0.996)] and small PICC diameter (1.9Fr, *p* = 0.003, OR (95%CI) = 3.936(1.578, 9.818)] were risk factors for PICC complications. Correction of malpositioned catheters was attempted and all succeeded in 9 eligible cases by using arm movements.

**Conclusion:**

The nurse-inserted PICC program in general pediatrics is feasible with a low rate of complications. PICC tip malposition and occlusion were two major PICC-related complications when low age and small catheter lumina were major risk factors. Furtherly, arm manipulation potentially is an easy and effective approach for correcting malpositioned newly-inserted PICC catheters.

## Key messages

The nurse-inserted PICC program in general pediatrics out of intensive care units is feasible, with a low rate of complications.

PICC tip malposition and occlusion were two major PICC-related complications in the nurse-inserted PICCs, and younger age and smaller catheter lumina were risk factors.

Arm manipulation, as an easily performed practice, is effective in correcting mal-positioned newly-inserted PICC catheters.

## Background

Obtaining peripheral venous access in pediatric patients with chronic disease for the purpose of infusing parenteral nutrition or stimulant drugs is often challenging for nursing professionals. A peripherally inserted central catheter (PICC) with its tip preferably in the vena cava allows for middle to long-term intravenous therapy, blood sampling, and reductions in repeated intravenous catheterizations [1, 2]. Though PICC insertion is a relatively convenient and effective technique, it can cause life-threatening complications such as catheter-related bloodstream infection [3, 4] and pericardial tamponade [5–8].

PICCs, inserted by nurses at the bedside, have been routinely used in the neonatal and pediatric intensive care units for decades in our hospital, a leading pediatric academic center in China. With increasing numbers of pediatric patients with chronic conditions referred from other hospitals, PICC insertion in pediatric sub-specialties other than intensive care units, such as the department of gastroenterology and pulmonology, was promoted. However, interventional pediatric radiologists inserting PICC under fluoroscopic guidance, which is common in developed countries, is not available yet in China. Therefore, a nurse-led PICC insertion program outside of the intensive care unit was established in the department of gastroenterology and pulmonology in Dec. 2015. Since then, there have been 169 patients who received PICC insertion in the department. The objective of this study was to share the experience of a nurse-inserted pediatric PICC program outside intensive care units, mainly focusing on PICC-related complications and risk factors.

## Patients and methods

### Study design, setting, and participants

This is a retrospective cohort study. All inpatients with PICC insertion in the department of gastroenterology and pulmonology at the Children’s Hospital of Fudan University from Dec. 2015 through Dec. 2019 were included in the study. The department is a leading pediatric referral center in China for the treatment of chronic pediatric gastrointestinal and lung diseases, such as infant onset inflammatory bowel disease, intractable diarrhea, infant chronic lung disease, and pneumonia with complications. Most of the admitted cases were referred from other hospitals across China. The study was approved by the Institutional Review Board of the Children’s Hospital of Fudan University.

The indications for PICC insertion in the department included: patients requiring intermediate- to long-term intravenous access for medications, fluid therapy, or parenteral nutrition; expected difficult peripheral IV catheter insertion during hospitalization. Contraindications included infection at the insertion site; damaged or thrombosed vessels caused by previous catheter insertions or repeated attempts; severe coagulopathy or thrombocytopenia. All PICCs performed in the department during the 4-year period were included in the current study.

### PICC practice

Informed consent was obtained from the parent or guardian. PICC insertion occurred in a therapeutic suite using a sterile technique and a topical anesthetic was applied by PICC nurses. All involved nurses need to get a 6-month training in PICC insertion, dressing, and troubleshooting prior to an independent performance, and they need to perform at least 10 PICCs per year to maintain qualifications. The insertion procedure and post-insertion maintenance were guided by institutional protocol. Bedside ultrasound was used in assessing the diameters of targeted veins and guiding needle insertion mainly in elder children with Fr 3.0 or 4.0 catheters, but not in infants with relatively smaller veins. The placement of the PICC tips was confirmed by x-ray after insertion with tips in the vena cava as the preferred position [1]. Of note, if PICC tip malposition occurred in upper limb insertion, a serial arm movements procedure as reported by Nadroo et al. was attempted to correct the malposition in eligible patients [9]. For PICCs inserted via basilic veins, the arm was abducted at the shoulder and the elbow extended as far as possible, then adducted at the shoulder and flexed at the elbow; for PICCs inserted via cephalic veins, the arm was adducted at the shoulder and extended at the elbow as far as possible, then the shoulder was abducted and the elbow flexed. After the movements, a repeat x-ray was performed within 24 hours to confirm the placement of the PICC.

### Data collection and definition

All data including patient gender, age, clinical diagnosis, indications for PICC, insertion procedure, post-insertion x-ray, PICC dwell time, and complications were extracted from a prospectively registered database. All clinical data related to the PICC were timely input into the database when the patient was in the hospital. PICC procedure time was recorded from entry into the procedure room to exit from the room after completion of the procedure. PICC tip placement was determined by radiography as either central or non-central. Reasons for PICC removal were categorized as planned if the therapy ended, otherwise categorized as unplanned removal. Complications of PICC were defined as following [10]: malposition, the inappropriate position of PICC tip after insertion; catheter occlusion, blockage of the catheter; dislodgment, accidental removal of PICC; fracture and fragmentation, catheter breakage; mechanical phlebitis, clinical signs of tenderness, erythema, and edema at insertion site; infection, systemic or insertion site bacteremia or fungi infection.

### Statistical analysis

Analyses were performed using SPSS 25 (SPSS, Inc., Chicago, IL). Patient characteristics were demonstrated as counts with proportions for categorical variables and median with interquartile range (IQR) for continuous variables. The comparison between patients with and without complications was conducted using Pearson chi-square or Fisher’s exact test for categorical variables and the Mann-Whitney U test for continuous variables. The variables with a *p*-value less than 0.1 were collected and used to study the risk factors for PICC-related complications by logistic regression analyses using multivariate models. PICC diameter choice was significantly dependent on age, therefore, PICC diameter and age were collinear variables and put in the model separately. A *p*-value< 0.05 was considered statistically significant.

## Results

### PICC and patient characteristics

There were 169 cases of patients with a median (IQR) age of 42(6, 108) months who received PICC insertion during the 4-year period. Inflammatory bowel disease (43/169, 25.4%) was the leading diagnosis in all cases followed by chronic diarrhea(18/169, 10.7%), upper gastrointestinal bleeding(18/169, 10.7%), pneumonia(15/169, 8.9%) etc. Requiring parenteral nutrition was the major indication for PICC insertion in 65.1% (110/169) cases followed by expected intravenous infusion requirement> 6 days (49/169, 29.0%) and difficult peripheral intravenous catheter insertion (10/169, 5.9%). For all PICC insertions, one puncture success rate was 52.7% (89/169); the total device days was 2859, with a median catheter retention duration of 12 (8, 20) days; 32.5% (55/169) received 1.9 Fr catheter; 43.2%(73/169) was inserted through a basilic vein.

### Complications of PICC and risk factors

Forty-seven onsets of complications occurred in 43 cases including 16 catheter malposition, 14 catheter occlusion, 9 mechanical phlebitis, 6 external breakages, 1 catheter-related bloodstream infection, 1 bleeding, and 1 limb edema. The total complication rate was 16.4 per 1000 catheter days. 56.3% (9/16) of malposition and 100% (14/14) of occlusion occurred in 1.9Fr cases. The complications of PICC were demonstrated in table 1.

**Table 1:** Complications of PICC and clinical outcomes

Complications	Cases (rate per 1000 catheter days) ^a^	Catheter diameter/Fr			Treated in place	unplanned removed/exchanged
		1.9	3.0	4.0		
Malposition	16(5.6)	9	2	5	16	0
Occlusion	14(4.9)	14	0	0	11	3
Mechanical phlebitis	9(3.1)	2	1	6	9	0
External breakage	5(1.7)	3	2	0	2	3
Catheter-related bloodstream infection	1(0.3)	0	1	0	0	1
Bleeding	1(0.3)	0	0	1	0	1
Impaired venous return	1(0.3)	0	1	0	0	1
Total	47(16.4)	28	7	12	38	9

^a^ Total catheter days = 2859

The comparison between patients with and without PICC complications demonstrated that age, procedure time, catheter diameter, and puncture attempts were potential risk factors for complications with a *p*-value less than 0.1(Table 2). As age and catheter diameter were collinear variables, two multivariate models were performed by logistic regression, with the results demonstrating that young age and small PICC diameter were risk factors for PICC complications (Table 3).

**Table 2:** Comparison of patients with and without PICC-related complications

Variables	Total *N* = 169	With complications *N* = 43	without complications *N* = 126	*P*-value
Sex-male — n (%)	97(57.4)	28(65.1)	70(55.6)	0.273
Age in months—Median (IQR)	42(6108)	9(2,50)	66(12,120)	<0.001
Age categories — *n* (%)				0.001*
1 month-1 year	59(34.9)	25(58.1)	34(27.0)	
~5 year	37(21.9)	9(20.9)	28(22.2)	
~10 year	40(23.7)	6(14.0)	34(27.0)	
~16 year	33(19.5)	3(7.0)	30(24.8)	
Total device days—Median (IQR)	12(8, 20)	13(10,24)	12(8,20)	0.433
Procedure time—Median (IQR) mins	40(30,80)	60(30,120)	40(30,60)	0.031
Puncture attempts — n (%)				
One	89(52.7)	16(37.2)	73(57.9)	0.061
Two	25(14.8)	8(18.6)	17(13.5)	
Three or more	55(32.5)	19(44.2)	36(28.6)	
Catheter diameter — n (%)				< 0.001
PICC 1.9Fr	55(32.5)	24(55.8)	31(24.6)	
PICC 3.0Fr	22(13.0)	6(14.0)	16(12.7)	
PICC 4.0Fr	92(54.4)	13(30.2)	79(62.7)	
Sites of insertion — *n* (%)				0.309*
Right arm	117(69.2)	25(58.1)	92(73.0)	
Left arm	31(18.3)	10(23.3)	21(16.7)	
Leg	11(6.5)	4(9.3)	7(5.6)	
Jugular vein	10(5.9)	4(9.3)	6(4.8)	

*Fisher’s exact test. *IQR *Interquartile range, *PICC* Peripherally inserted central catheter

**Table 3:** Risk factors for PICC-complications in pediatric cases identified by logistic regression

Risk factors	B	Wald	*P*-value	Odds Ratio [95%]
Model 1^a^				
Age (month, continuous)	−0.013	8.114	0.004	0.987[0.978, 0.996]
Procedure time (minute, continuous)	0.002	0.199	0.655	1.002[0.993, 1.012]
Puncture number				
One	−0.284	0.345	0.557	0.753[0.293, 1.939]
Two	0.3	0.271	0.603	1.350[0.436, 4.183]
Three or more	Reference	.	.	.
Model 2^b^				
Catheter diameter				
1.9 Fr	1.37	8.632	0.003	3.936[1.578, 9.818]
3.0 Fr	0.867	2.236	0.135	2.380[0.764, 7.417]
4.0 Fr	Reference	.	.	.
Procedure time (minute, continuous)	0.002	0.221	0.638	1.002[0.993, 1.012]
Puncture attempts				
One	−0.267	0.297	0.586	0.765[0.293, 2.001]
Two	0.413	0.496	0.481	1.511[0.479, 4.766]
Three or more	Reference	.	.	.

^a^Model 1 variables included age, procedure time, and puncture attempts

^b^Model 2 variables included catheter diameter, procedure time, and puncture attempts

### PICC malposition correction

**Fig. 1:**
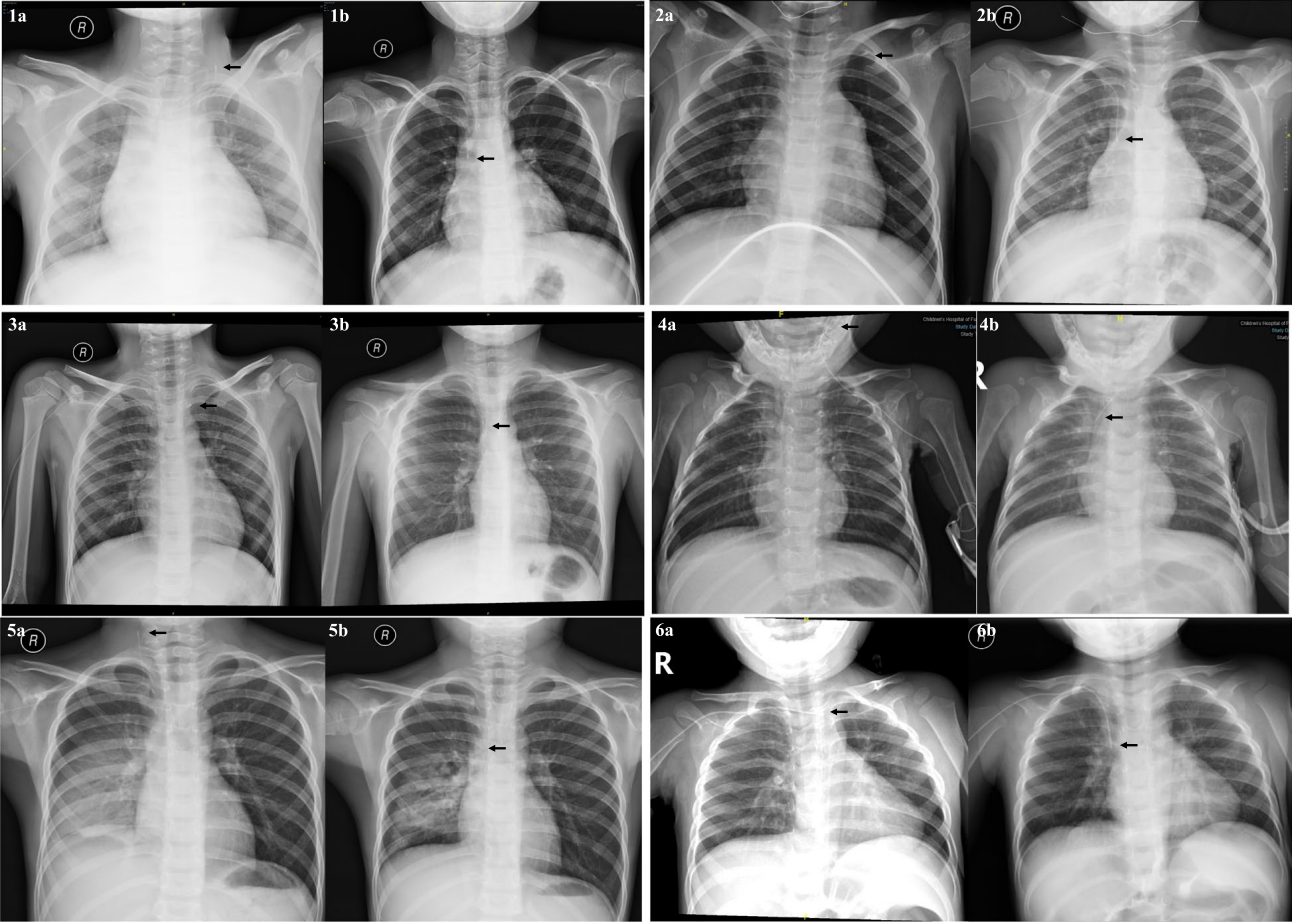
X-ray images of PICC before(a) and after arm movements(b). **1a/1b**, 13-year boy, right basilic vein insertion, 4.0 Fr catheter, the tip was in the right jugular vein before arm movements. **2a/2b**, 6-year girl, right basilic vein insertion, 4.0 Fr catheter, the tip was in the left subclavian vein before arm movements. **3a/3b**, 5-year boy, right cephalic vein insertion, 4.0 Fr catheter, the tip was in the left subclavian vein before arm movements. **4a/4b**, 5-month boy, left axillary vein insertion, 1.9Fr catheter, the tip was in the left jugular vein before arm movements. **5a/5b**, 7-year boy, right cephalic vein insertion, 3.0 Fr catheter, the tip was in the right jugular vein before arm movements. **6a/6b**, 20-month boy, right basilic vein insertion, 3.0 Fr catheter, the tip was in the left brachiocephalic vein before arm movements. Tips of all PICCs were in the superior vena cava after arm movements

Of 16 incidents of PICC malposition, 9 cases eligible for arm movement were successfully corrected by arm movements. The typical x-ray images before and after the maneuver were demonstrated in fig. 1 (6 cases were demonstrated, while the other 3 cases were reported previously [11]).

## Discussion

This study reported the experience of a nurse-inserted PICC program in a pediatric sub-specialty outside intensive care units in a tertiary children’s hospital from China. Of all cases, 83.4%(141/169) were from the department of gastroenterology with inflammatory bowel disease as the most common diagnosis while 16.6%(28/169) were from pulmonology with pneumonia as the most common diagnosis. The age range of cases was from 1 month to 16 years old. PICCs inserted during the study had catheter diameters ranging from 1.9Fr to 4.0Fr. Regarding the age range of patients, PICC insertion in such a pediatric sub-specialty seems more challenging than that in neonatal intensive care units, where age and body weight were more uniform.

Our study demonstrated the feasibility of PICC insertion in pediatric sub-specialties apart from intensive care units which are consistent with previously published articles. For example, Piper et al. reported that, for infants with intestinal failure, PICCs offer an advantage over central venous catheters with a low rate of catheter-related bloodstream infection and venous thrombosis. The study recommended PICCs for infants with intestinal failure requiring parenteral nutrition [12]. And PICC usage was increasing annually in other pediatric centers [13]. In China, PICC use in pediatric sub-specialties other than oncology and intensive care units is underdeveloped. Since interventional pediatric radiologists inserting PICCs under fluoroscopic guidance, which is common in developed countries, is not widely available yet in China. The nurse-inserted PICC program reported in this study is a feasible alternative model meriting introduction to other pediatric subspecialties.

In our study, we reported a low PICC-related complication rate of 16.4 per 1000 catheter days. The result is similar to other reports. In a review, Westergaard et al. reported the overall rates of complications in pediatric populations ranging from 1.11 to 19.3 per 1000 catheter days [14]. Of all complications in our cases, only 19.1%(9/47) required removal or replacement of the PICC, the rate was much lower than one-third as reported in the previous publications [15]. Therefore, only a small proportion of complications in cases included in this study could be classified as severe.

We found that malposition was the leading complication in our cases, however, 93.8%(15/16) of malposition occurred just after insertion; only one occurred as a result of patient behavior (severe cough). And most of the primary malposition occurred in 1.9Fr cases, ultrasound not being used for guiding PICC procedure in such a low age group potentially delayed the detection of malposition. PICC occlusion was the second most common complication, all occurring in cases with 1.9Fr lumen PICCs. Daily flushing of the catheter with saline or a heparin solution(50-100 U/ml) is a potentially effective approach to preventing PICC occlusion [14]. Besides malposition and occlusion, we reported a much lower rate of catheter-related bloodstream infection(0.3 per 1000 catheter days) than those reported in previous studies (from 1.4 to 2.0 per 1000 catheter days) [16, 17]. Standard PICC insertion and dressing practice guided by institutional protocols and early removal of unnecessary catheters are essential in preventing PICC-related bloodstream infection. Furtherly, we ascertained that the risk factors for PICC complications were low age and small PICC lumina which is similar to other study [18]. As mentioned above, promoting bedside ultrasound usage and practicing daily flushing using normal saline or low concentration heparin warrant further investigation in preventing small lumen catheter-related complications in young children.

Of 16 malposition, 9 were corrected by arm movement. Correct PICC tip position by arm manipulation has been reported previously. Nadroo et al. reported that arm movements significantly affect the position of the tip of the PICCs in neonatal cases. For catheters that were placed in the basilic vein, simultaneous shoulder adduction and elbow flexion caused the greatest movement toward the heart(15.11 ± 1.22 mm)^9^. Another adult study reported that there was a large amplitude of PICC tip position change with the depth of inspiration and arm position [19]. Since arm movement significantly alters PICC tip position, there were studies using arm movement for correcting malposition. Nadroo et al. described the maneuver for correcting PICC malposition and approved the effectiveness [9]. The maneuver was used in this study. Besides arm movement, a high-flow flush technique was another approach to malposition correction studied in previously published studies with a success rate of approximately 70% [20]. Of note, arm movement and high-flow flush are only alternative approaches for PICC malposition correction while radiologic intervention is the first or only choice in certain complicated cases [21].

Our study comprehensively reported the experience of PICC practice in infants and children outside intensive care units from a single tertiary pediatric center. All clinical data were collected prospectively, therefore, the data accuracy was high. Risk factor analyses for complications and malposition correction techniques provided a valuable reference for counterparts. Our study has limitations as well. Firstly, in analyzing risk factors for complications, some risk factors reported in previous studies [22, 23], such as dual lumen catheters, non-central position of the catheter tip, were not identified due to small sample size (only 2 non-central cases) and only single lumina catheters used in our practice. Secondly, Rastogi et al. reported that a mal-positioned PICC potentially could be corrected spontaneously [24]. Arm movement was approved effective in malposition correction in our descriptive study, however, without non-intervention control, the necessity of proactive correction by arm movement warrants further comparison studies.

Even though the complication rate in overall cases was not remarkably high, there is still large room for improvement. The following approaches could be carefully reviewed in future cases: ultrasound for guiding PICC insertion [25, 26], routine daily heparin flushing in 1.9Fr cases [27], novel approaches for guiding PICC insertion, such as intracavitary electrocardiogram etc. [28].

## Conclusions

The nurse-inserted PICC program in general pediatrics is feasible with low complication rate. PICC tip malposition and occlusion were two major PICC-related complications with low age and small catheter lumina as risk factors. Arm movement potentially is an easy approach for correcting malpositioned PICC catheters.

## Data Availability

The data that support the findings of this study are available from the corresponding author, Jianguo Zhou.

## Abbreviations

PICC: Peripherally inserted central catheter
